# Association between hypoglycemia and poor clinical outcomes in hospitalized non-diabetic patients with liver cirrhosis:– a narrative review

**DOI:** 10.3389/fmed.2025.1541471

**Published:** 2025-05-16

**Authors:** Rohit Govindarajan, Jiancong Chen, Kunsong Zhang, Wenjie Hu, Dan Xu, Ming Kuang

**Affiliations:** ^1^General Practice Research and International Collaboration, Faculty of Health Sciences, Curtin Medical School, Curtin University, Perth, WA, Australia; ^2^Department of Hepatobiliary Surgery, The First Affiliated Hospital, Sun Yat-sen University, Guangzhou, China; ^3^Department of Medical Education, The First Affiliated Hospital, Sun Yat-sen University, Guangzhou, China; ^4^Centre for Clinical Research and Education, Faculty of Health Sciences, Curtin School of Population Health, Curtin University, Perth, WA, Australia

**Keywords:** hypoglycemia, chronic liver disease, cirrhosis, complications, clinical guidelines

## Abstract

Hypoglycemia is rarely highlighted as a complication that requires close monitoring in patients with chronic liver disease, despite substantial evidence of its occurrence in cirrhotic patients. This narrative review aims to evaluate whether hypoglycemia in liver cirrhosis patients, irrespective of diabetes status, exacerbates complications and warrants targeted management strategies. Our analysis reveals that hypoglycemia is prevalent in cirrhotic patients and is associated with increased mortality and complications compared to normoglycemic patients. Although literature in this topic is limited, our review suggests that early identification of high-risk liver disease patients and the implementation of novel, clinically relevant strategies to minimize hypoglycemia may improve clinical outcomes and health-related quality of life as well as reduce morbidity and mortality. Further research will be required to validate thesel strategies.

## Introduction

Hypoglycemia is commonly discussed in relation to diabetic patients, particularly those using oral hypoglycemic agents or insulin therapy. However, its occurrence in patients with liver disease is also notable. This can be attributed to various factors, including the liver’s critical role in glucose metabolism. Despite this, the significance of hypoglycemia in chronic liver disease is rarely addressed, and international clinical guidelines for cirrhosis often overlook its management. This narrative review aims to bridge this gap by critically reviewing the current literature to elucidate the relationship between hypoglycemia and liver cirrhosis. We aim to assess whether protocol implementation to identify and prevent hypoglycemia could improve patient outcomes, irrespective of diabetes status. By focusing on this unexplored aspect of liver disease management, we seek to highlight gaps in the existing literature and contribute to better clinical practice and improved care for patients with liver cirrhosis.

## Methods

The authors conducted an extensive literature review in Ovid Medline, Ovid Embase, CINHL, Web of Science, and PsychINFO (OVID), employing the following Medical Subject Headings (MeSH) terms: hypoglycemia, chronic liver disease, cirrhosis, acute on chronic liver disease and decompensated cirrhosis. The review identified 222 studies related to the association between hypoglycemia and chronic liver disease. After scanning the titles and abstracts to remove duplicates, case reports, and editorial comments, 12 publications were selected for full-text screening to explore the significance of hypoglycemia in hospitalized patients with chronic liver disease. The Oxford Equator PRISMA checklist was applied to ensure that the review adhered to evidence-based standards for narrative reviews. This checklist, an internationally recognized guideline, ensures transparency, integrity, and validity in reporting systematic and narrative reviews.

### Overview

Hypoglycemia is defined by serum glucose levels typically below 3.9 mmol/L (1). It is a well-known and feared complication in the management of diabetic patients (2). Severe hypoglycemia refers to any hypoglycemic event that requires external assistance for recovery (3). It is associated with falls, neurological disease, cardiovascular events, cognitive impairment, and increased mortality (4).

Cirrhosis is a chronic progressive, end-stage liver disease characterized by the replacement of normal liver tissue with fibrous scar tissue, which disrupts the liver’s normal structure and function (5). This includes alterations in key hepatic metabolic processes, such as gluconeogenesis and glycogenolysis, both of which normally contribute to maintaining higher serum glucose levels (6).

During a 2–6 h fast, hepatocytes initiate glycogenolysis, breaking down stored glycogen to release glucose for energy (2). In a state of prolonged fasting, hepatocytes utilize substrates like lactic acid, amino acids, and glycerol to synthesize glucose through gluconeogenesis (2). However, abnormal liver metabolism or cellular damage impairs the liver’s ability to regulate blood glucose. Approximately 5%–7% of cirrhotic patients progress to decompensated cirrhosis each year (7). Decompensated cirrhosis is an advanced stage of the disease, marked by severe complications such as hepatic encephalopathy, ascites, and/or variceal bleeding (7).

#### Is hypoglycemia prevalent among cirrhotic patients without diabetes

Hypoglycemia is frequently observed in cirrhotic patients. Singh et al. (8) reported hypoglycemia in 67% of cirrhotic patients without diabetes, while Noul et al. (9) found it in 50% of individuals hospitalized for septicemia (8, 9), none of whom were on hypoglycemic agents (9). Majeed et al. (10), through a cross-sectional study, also found hypoglycemia in 51.2% of liver cirrhosis patients after excluding those with diabetes, although significant grammatical errors in the study affected its reliability. While less pronounced, Gladys-Oryhon et al. (11) still observed hypoglycemic events in 34.7% of non-diabetic cirrhotic inpatients .

Several factors contribute to the high prevalence of hypoglycemia in cirrhotic patients, including (i) persistent cachexia, especially in decompensated cirrhosis, (ii) reduced hepatocyte mass, leading to decreased gluconeogenic capacity, (iii) sarcopenia, which limits the availability of amino acids necessary for hepatic gluconeogenesis, and (iv) comorbid conditions such as congestive heart failure, chronic pancreatitis with glucagon deficiency, chronic kidney disease, and hepatorenal syndrome. As the liver function deteriorates, the incidence of fasting hypoglycemia rises significantly, indicating the liver’s inability to regulate insulin glucose homeostasis in chronic disease (12).

#### What do current guidelines suggest regarding hypoglycemia in cirrhotic patients?

Whilst continual glucose monitoring is strongly emphasized in hospitalized diabetic patients, regardless of cirrhosis, the European Association for the Study of the Liver (EASL) clinical practice guidelines do not mention tight glucose control for patients admitted with decompensated cirrhosis (13). Similarly, guidelines for compensated liver cirrhosis, such as those by Yoshiji et al. (14) and the British Society of Gastroenterology, do not address hypoglycemia management in non-diabetic cirrhotic patients (15). Whether hypoglycemia should be a concern in cirrhotic patients admitted for reasons other than decompensation, warrants further review.

Another important question is whether avoiding hypoglycemic episodes in hospitalized cirrhotic patients, decompensated or otherwise, could improve outcomes. The only guideline we found that explicitly addresses this is from the American Society of Critical Care Medicine, which states that preventing hypoglycemia in ICU patients with acute-on-chronic liver disease can improve outcomes (16). However, this recommendation is limited to ICU patients, and no clear guidance exists for managing hypoglycemia in non-ICU cirrhotic inpatients, whether admitted with decompensation, acute-on-chronic disease, or other conditions.

Although hypoglycemia management is not specifically included in liver disease guidelines, the American Society of Parenteral and Enteral Nutrition and the European Society for Clinical Nutrition and Metabolism recommended that patients with severe liver dysfunction consume extra nighttime meals to prevent hypoglycemia during temporary fasting.

However, these guidelines do not elaborate on whether this practice should be generalized to all cirrhotic patients (17, 18).

The current literature clearly indicates that hypoglycemia is common amongst cirrhotic patients, irrespective of their diabetes status. To address the significance of identifying hypoglycemia, we reviewed the available evidence. Our goal was to determine whether hypoglycemia is linked to poor outcomes and whether preventing these hypoglycemic events could lead to improved patient outcomes.

#### Increased adverse outcomes in hypoglycemic patients with cirrhosis admitted to hospital

Obeidat et al. (19) conducted a retrospective study involving 1,778,829 in-patients with cirrhosis, excluding those with diabetes. The study revealed that in-patient mortality was significantly higher than in the hypoglycemia group compared to the non-hypoglycemia group of cirrhotic patients (OR 6.8; CI 95% 6.4–7.24, *P*-value < 0.001) (19). Additionally, patients in the hypoglycemic group had a longer and more complicated hospital stay, with increased likelihood of vasopressor use, mechanical ventilation, cardiac arrest, and ICU admission (19).

Similarly, Hung et al. (20) reported a 30 days mortality rate of 30.2% in the hypoglycemic group, compared to 7.4% in the non-hypoglycemic group (*P* < 0.001) among hospitalized cirrhotic patients without diabetes. This study further found that the 30 days mortality was even higher in patients with hypoglycemia and hepatocellular carcinoma (HCC), with a hazard ratio of 6.11 (95% CI 4.40–8.49, *P* < 0.001) compared to 4.96 (95% CI 4.05–6.08, *P* < 0.001) for patients without either condition (20).

Although many studies have demonstrated poor outcomes in cirrhotic patients with hypoglycemia, the benefit of preventing hypoglycemia remains unclear. Additionally, there are no current consensus guidelines for monitoring glucose levels in cirrhotic patients. It is also unclear whether hypoglycemia prevention should be applied universally to all cirrhotic patients or targeted specifically to higher-risk groups. Future studies are needed to address these questions and potentially improve the clinical outcome of cirrhotic patients.

#### Increased adverse outcomes in hypoglycemic patients with cirrhosis admitted in hospital with decompensated cirrhosis

The study by Pfortmueller et al. (21) explored the relationship between hypoglycemia on admission in patients presenting to the emergency department with acutely decompensated cirrhosis. The study found that patients with hypoglycemia were significantly more likely to be admitted to the ICU compared to normoglycemic patients (20.4% vs 10.3%, *P* < 0.011). Additionally, the hypoglycemic group had a higher mortality rate rather than the normoglycemic group (28.6% vs 10.3%, *P* < 0.049), with an estimated survival of 36 days compared to 54 days for the normoglycemic group (*P* < 0.007) (21).

The study also showed a significant association between hypoglycemia and hepatorenal syndrome in decompensated cirrhosis, which may contribute to the increased mortality in the hypoglycemic group (21, 22). Olson et al. (22) highlighted that there are currently no recommendations to treat hypoglycemia in these patients on admission, despite clear evidence of worse prognosis and clinical outcomes. Therefore, the author suggests evaluating whether prophylactic glucose administration could improve clinical outcomes in hypoglycemic patients (22). Future studies should investigate the potential benefit of preventing hypoglycemia in cirrhotic patients through strategies such as prophylactic glucose and nighttime carbohydrate consumption (18, 22).

#### Increased adverse outcomes in hypoglycemic patients who were admitted to hospital with acute on chronic liver failure

Acute-on-chronic liver failure is a syndrome characterized by the acute deterioration of liver function in patients with pre-existing chronic liver disease, often triggered by factors such as infection, gastrointestinal bleeding, or alcohol consumption (23). A study by Yang et al. (24) involving 218 patients with acute-on-chronic liver failure found hypoglycemia in 45.41% of cases. Hypoglycemia was associated with significantly higher 90 days mortality compared to non-hypoglycemic patients (72.73% vs 48.74%, *P* < 0.001).

The increased mortality was further reflected in additional findings, with hypoglycemic patients showing higher levels of AST (264 vs 216), total bilirubin (379 vs 308), and MELD score (31 vs 25), consistent with the findings of Olsen et al. (22). The analysis of risk factors for hypoglycemia in these patients revealed that liver cirrhosis (OR 5.16) and higher MELD score (OR 1.29) were significant risk factors for hypoglycemia (24). Conversely, higher fibrinogen levels appeared to reduce the risk of hypoglycemia (OR 0.17) (24).

These findings suggest that hypoglycemia may serve as an early indicator of acute-on-chronic liver failure, as evidenced by elevated AST, INR, creatinine, and bilirubin level in hypoglycemia patients, which were not observed in normoglycemic individuals (22).

These findings not only reinforce the evidence of increased adverse outcomes in hypoglycemic cirrhotic patients but also suggest a potential pathway for stratifying and identifying the most at-risk cohorts. This stratification could be based on various criteria, including AST, bilirubin, INR, creatinine, MELD scores and fibrinogen levels (22, 24). Further exploration may provide insights into how stratification can be applied to ensure that high-risk patients are promptly identified and closely monitored.

#### Hypoglycemia among cirrhosis patients as a predictor of bacteremia and septicemia?

In addition to the increased mortality seen in cirrhotic patients experiencing hypoglycemia, a study by Yedidya et al. (25) demonstrated that hypoglycemia is predictive of bacteremia. Among 1,274 cirrhosis admissions, glucose levels below 5.6 mmol/L increased the likelihood of subsequent bacteremia, even in normothermic patients (25). This study suggests that hypoglycemia could be used as a clinical predictor for bacteremia, raising the question of whether prophylactic antibiotic therapy may be warranted in cirrhotic patients with hypoglycemic events. There is some supporting evidence that prophylactic antibiotics might reduce acute exacerbations of chronic liver diseases (25).

Another study by Nouel et al. (9) found that 50% of cirrhotic patients with septicemia had asymptomatic hypoglycemia. The study also noted that hypoglycemia is commonly seen in cirrhotic patients with septic shock, potentially secondary to endotoxemia. Tanveer et al. (26) further established that hypoglycemia in decompensated cirrhotic patients was consistently associated with septicemia.

Ultimately, future studies are needed to determine whether early identification of hypoglycemia could serve as a predictor for septicemia and justify the use of prophylactic antibiotics or further investigations, such as blood cultures, to improve patient outcomes.

#### Is there a clear protocol or recommendation for managing hypoglycemia among cirrhosis patients to improve clinical outcomes?

On the balance of the current literature review, a few recommendations can be clearly summarized as follows:

1. Preventing hypoglycemia in ICU patients with acute-on-chronic liver disease and decompensated liver cirrhosis.
2. Preventing hypoglycemia in cirrhotic patients with severe liver dysfunction.
3. Preventing hypoglycemia in cirrhotic patients with acute-on-chronic liver failure.
4. Preventing hypoglycemia in cirrhotic patients with hepatorenal syndrome.
5. Hypoglycemia can be used as a clinical predictor for bacteremia and septicemia, and prophylactic antibiotics can be used in cirrhotic patients to reduce acute exacerbations of chronic liver diseases.

These recommendations are targeted specifically to higher-risk groups without an overarching statement to declare that hypoglycemia prevention can be applied universally to all cirrhotic patients due to the study’s small sample size and methodological limitations. Future studies with large sample sizes and improved methodological design are needed to address these questions and limitations as well as the related study biases for the potential improvement of clinical outcomes with cirrhotic patients.

## Conclusion

Although current chronic liver disease management guidelines rarely address hypoglycemia in non-diabetic patients, this review highlights its significance in hospitalized patients with liver disease. There is limited but compelling evidence linking hypoglycemia to poor clinical outcomes in liver disease patients, whether admitted with another condition, decompensated cirrhosis, or acute-on-chronic liver disease, independent of diabetes (9, 18–26).

Given the scarcity of studies on hypoglycemia in cirrhotic patients, there is significant potential for multi-center trials to explore these uncertainties and inform updates to existing management guidelines. This includes developing tools that utilize clinical parameters such as MELD score, AST, bilirubin, and others to (1) identify and stratify patients at high risk for hypoglycemia and (2) prevent hypoglycemic events, thereby reducing associated poor outcomes such as mortality, ICU admissions, and complications like septicemia (22–24).

While hypoglycemia is clearly associated with poor clinical outcomes, it remains unclear whether prevention strategies-such as prophylactic glucose administration, nighttime carbohydrate intake, and early identification -will improve patient outcomes (18, 22).

Moreover, the interaction between septicemia, cirrhosis, and hypoglycemia raises important questions about the potential benefits of (1) administering prophylactic antibiotics and (2) conducting prompt blood cultures when hypoglycemia is detected, or when a cirrhotic patient is identified as being at high risk for hypoglycemic events (25, 26).

Furthermore, the mechanism of hypoglycemia in cirrhotic patients has been shown to be related to reduced hepatic glycogen stores in patients with liver cirrhosis (27). The conclusion of this study is that patients with alcoholic or biliary cirrhosis have decreased hepatic glycogen stores per volume of hepatocytes and per liver, and decreased glucokinase activity may be the important underlying mechanism (27). Identification of the mechanism of hypoglycemia with cirrhotic patients will be one of the priorities for future research.

Table 1 has a detailed summary of the characteristics, main results and possible bias of the included studies for discussion and analysis in this mini review to raise our research questions for overcoming the above-mentioned limitations. Addressing these questions through future research could significantly improve the management and clinical outcomes of hospitalized patients with liver cirrhosis, which may translate into improved quality of life, reduced morbidity, or even mortality.

**TABLE 1:** Characteristics, main results, possible bias of included studies.

References	Study design	Study population	Purpose of study	Main results	Possible bias
Singh et al. (8)	Cross sectional study	100 patients with liver cirrhosis > 12 years of age at Liaquat University Hospital in Hyderabad, Pakistan.	To identify hypoglycemia among cirrhotic patients without diabetes.	Hypoglycemia was observed in 67% of patients with liver cirrhosis.	Selection bias – patients recruited from a single hospital. Sampling bias – *n* = 100, limits generalizability. Measurement bias – glucose levels measured using glucometers, which may be less accurate that laboratory testing. A single-time-point assessment may miss intermittent or nocturnal hypoglycemia. Lack of confounding variable control – nutritional status, infection status, comorbid conditions, are not controlled for in this study, despite potentially influencing glucose levels. Grammar – grammatical errors throughout article may reduce clarity.
Nouel et al. (9)	Observational prospective cohort study	30 patients with cirrhosis and septicemia in Hopital Beaujon, France.	To identify relationship between hypoglycemia, septicemia and mortality among cirrhotic patients.	50% of cirrhosis patients with septicemia had hypoglycemia. 100% of patients with hypoglycemia developed circulatory failure (septic shock), compared to 0% of normoglycemic patients. 11/15 of hypoglycemia patients died within 24–48 h due to septic shock; 3/15 died later due to liver failure; only 1/15 survived. (Mortality rate: 93%). Among normoglycemic patients, 10/15 died, none due to septic shock (mortality rate: 67%).	Sampling bias – *n* = 30, reduces statistical power. Measurement bias – glucose measured only once daily, which may potentially omit intermittent or nocturnal hypoglycemia. Confounding – severity of underlying liver disease, could be responsible for hypoglycemia and development of septic shock. The study doesn’t stratify according to degree of liver cirrhosis. Degree of sepsis is also not commented on, as patients with more prominent infections may be at risk of both shock, mortality and hypoglycemia. Lack of control group – no comparison to cirrhotic patients without sepsis or septic patients without cirrhosis, limiting causal conclusions.
Majeed et al. (10)	Cross sectional study.	84 patients in Mayo Hospital, Lahore, Pakistan, who were aged 16–75 with liver cirrhosis and non-diabetic.	To identify hypoglycemia among cirrhotic patients without diabetes.	Hypoglycemia was observed in 51.2% with liver cirrhosis and who didn’t have diabetes. There was no correlation between severity of cirrhosis (as per Child Pugh score) and hypoglycemia.	Selection bias – single hospital. Sampling bias – *n* = 84, which limits statistical power. Measurement bias – use of glucometer less accurate than laboratory measurement. Single time-point may miss fluctuations in glucose levels. Confounding variables such as nutritional status, medications, comorbidities were not controlled.
Gladys-Oryhon et al. (11)	Retrospective chart review.	101 non-diabetic cirrhotic patients from a tertiary care hospital. Mean age 62 years.	To identify hypoglycemia among cirrhotic patients without diabetes.	22.8% of patients with cirrhosis and no diabetes, experienced hypoglycemia. Only 35% (35/101) had routine point of care (POC) glucose monitoring.	Selection bias – single center study. Information bias – retrospective design depends on accuracy of medical records. Confounding – no adjustment for severity of liver disease, nutritional status, infections, or medications. Detection bias – limited POC monitoring likely led to underestimation of hypoglycemia.
Honda et al. (12)	Cross sectional study.	105 patients with chronic liver disease with type 2 diabetes mellitus.	Aimed to identify hypoglycemia in cirrhotic patients with type 2 diabetes.	CGM was useful for detecting asymptomatic nocturnal hypoglycemia and undetected postprandial hypoglycemia. 22% of patients had nocturnal hypoglycemia.	Lack of control group of healthy individuals or chronic liver disease patients without diabetes, making it hard to generalize the results. Selection bias – *n* = 105, relatively small. Study was limited to T2DM patients, hence did not explore glycemic variability in non-diabetic patients.
Obeidat et al. (19)	Retrospective cohort study.	31,615 cirrhotic patients aged 18 or over, without diabetes were identified from the National Inpatient Sample database in the United States, from 2016 to 2019.	Aimed to analyze the impact that hypoglycemia had on patients with liver cirrhosis and without diabetes.	In-hospital mortality was significantly higher in the cirrhosis patient group with hypoglycemia (adjusted OR: 6.8). Other complications like mechanical ventilation (aOR: 5), vasopressor use (aOR: 4.33), cardiac arrest (aOR: 4.97) and ICU admissions (aOR: 5.09) were more frequent in the hypoglycemia patient group. Hypoglycemia was associated with longer length of stay (7.79 days vs 6.2 days in non-hypoglycemic group).	Use of ICD-10 codes for classification of cirrhosis and hypoglycemia may not be accurate or specific, leading to misclassification of patient’s conditions. Confounding bias – the study did control variables such as age, gender, race, comorbidities but did not factor in nutritional status, liver disease etiology or medications. Due to retrospective design, a definitive causality between cirrhosis, hypoglycemia and in-hospital complications cannot be established.
Hung et al. (20)	Retrospective cohort study.	636 cirrhotic patients without diabetes mellitus who presented with hypoglycemia from the Taiwan National Health Insurance Database (2010–2013).	To assess the effect of hypoglycemia at admission on 30 days mortality.	30 days mortality: 30.2% in hypoglycemia group vs 7.4% in non-hypoglycemia group. Adjusted hazard ratio: 4.96 for hypoglycemia; 6.11 when combined with HCC.	Use of ICD-9 codes for classification of cirrhosis and hypoglycemia may lead to diagnostic inaccuracies. Selection bias – may exclude patients with undetected hypoglycemia on admission. Confounding bias – the study did control variables of age, sex and comorbidities, however, other factors such as degree of liver function, medications, nutritional status were not controlled. The study claims that mortality is higher in hypoglycemia group with HCC, but there is no clarification on stage of HCC, as this can be a confounder affecting mortality independent of hypoglycemic state. Measurement bias – study did not clarify the definition of hypoglycemia. There may also be underreporting of hypoglycemia, especially if blood glucose levels not measured routinely in cirrhosis patients presenting to hospital. The patient demographics may not be generalizable to populations outside of Taiwan.
Pfortmueller et al. (21)	Retrospective cohort study.	312 patients aged 16 years and over, admitted into the Emergency Department of Inselspital, Bern University Hospital, Switzerland, between 1 Jan 2002 and 31 Dec 2012, with a primary diagnosis of acute decompensated liver cirrhosis. Patients identified using medical database software (Qualicare Office).	Study aimed to identify rate of glucose disturbance and outcomes associated including ICU admission and mortality in patients presenting with acute decompensated liver cirrhosis.	28.5% of patients experiences glucose disturbances; 15.7% hypoglycemia, 12.8% hyperglycemia. In-hospital mortality; 28.6% in hypoglycemic group vs 7.5% in hyperglycemic group vs 10.3% in normoglycemic group. Survival analysis indicated hypoglycemic group had lower survival (36 days) compared to normoglycemic (54 days) or hyperglycaemic (45 days) groups. ICU admissions were more likely in the hypoglycemia group. 20.4% of hypoglycemic patient’s vs 10.8% hyperglycemic patients vs 10.3% normoglycemic patients, were admitted to ICU.	Use of a database inherently has limitations and there may reporting bias present as a result. Detection bias – since serial glucose measurements were not performed, the true detection of hypoglycemia may be underestimated. Confounding – although the study accounted for age, sex, liver disease extent (using Child Pugh classification), and etiology of cirrhosis, it didn’t factor in medications, nutritional status and infection state.
Yang et al. (24)	Retrospective cohort study	218 patients diagnosed with acute on chronic liver failure. Study was conducted at First Affiliated Hospital of Xi’an Jiaotong University, China, between Jan 2019 and Aug 2021.	Aimed to identify risk factors for hypoglycemia and the impact of hypoglycemia on 90 days mortality.	Risk factors for hypoglycemia were liver cirrhosis (OR 5.16), a higher MELD score (OR 1.29), higher Fibrinogen (FIB) levels (OR 0.17). 45.41% of patients with acute on chronic liver failure had hypoglycemia. 90 days mortality was 72.73% in hypoglycemia group vs 48.74% in non-hypoglycemia group. After adjustment for hepatic encephalopathy, MELD score, and cirrhosis, hypoglycemia remained an independent risk factor for 90 days mortality (OR = 8.72).	Retrospective design – using historical data has its inherent bias, and potentially incomplete or missing records may result in errors. Generalizability may be limited as the population is from a single hospital only. Confounding – although the study adjusted for hepatic encephalopathy, cirrhosis, MELD scores, there may still be confounding variables such as medications, nutritional status, which may influence hypoglycemia and mortality. The study identified hypoglycemia as a risk factor for mortality, however, it does not investigate whether treatment of hypoglycemia may reduce mortality.
Yedidya et al. (25)	Retrospective case-control	1,274 patients aged 18 and over admitted with cirrhosis who had blood culture results. University of Pennsylvania Health System.	Aimed to identify relationship between hypoglycemia in cirrhotic patients and bacteremia.	52.7% of blood cultures were positive for bacteremia. 10.1% of patients with positive blood cultures had hypoglycemia 24–72 h prior, compared to 6.1% in those with negative blood cultures. Minimum glucose 24–72 h prior to blood culture result was a significant predictor for blood culture positivity. Glucose level < 100 mg/dL increased probability of positive blood culture (OR 1.89 for 50 mg/dL vs 100 mg/dL).	Use of ICD-9 codes for classification may not be accurate or specific, potentially leading to misclassification of patient’s conditions. Selection bias – single healthcare system used, may not be fully representative of generalized population. Information bias due to inherently being a retrospective study, and hence inaccuracies or missing data in the records could be present, including exact time of glucose measurement and culture results.
Tanveer et al. (26)	Cross sectional study	84 patients diagnosed with liver cirrhosis without diabetes. Study conducted in Department of Medicine, Mayo Hospital, Lahore, Pakistan.	Aimed to identify hypoglycemic patients.	51.2% had hypoglycemia.	Selection bias – single hospital. Findings may not generalize to greater population. Study only included outpatients and excluded hospitalized patients with cirrhosis. Information bias – using glucometer may have inaccuracies. Confounding bias – multiple factors such as medications, comorbidities, lifestyle factors do not seem to be adjusted in this study.
Krahenbuhl et al. (27)	Cross sectional study	Patients undergoing liver surgery. 17 cirrhotic patients (nine alcoholic cirrhosis and eight biliary cirrhosis), 14 control patients underdoing liver surgery but without cirrhosis.	Aimed to identify glycogen content and mRNA expression of glycogen metabolism related enzymes in control vs cirrhosis patients.	Cirrhotic patients had significantly lower hepatic glycogen content compared to control. Hepatic mRNA expression of glycogen metabolism-related enzymes was approximately 50% lower in cirrhosis patients compared to control.	Selection bias – sample size small with total 31 patients. Control group may not be representative of general population without liver disease. Measurement bias – the methods used for glycogen quantification may have errors from technical factors. Confounding – alcoholic and biliary cirrhosis may have different pathophysiology that dictates glycogen storage independent to liver cirrhosis. There is limited reporting on clinical outcomes, which creates a query regarding the significance that reduced glycogen stores may have in cirrhotic patients.
